# Loss from Treatment for Drug Resistant Tuberculosis: Risk Factors and Patient Outcomes in a Community-Based Program in Khayelitsha, South Africa

**DOI:** 10.1371/journal.pone.0118919

**Published:** 2015-03-18

**Authors:** Sizulu Moyo, Helen S. Cox, Jennifer Hughes, Johnny Daniels, Leigh Synman, Virginia De Azevedo, Amir Shroufi, Vivian Cox, Gilles van Cutsem

**Affiliations:** 1 Médecins sans Frontières (MSF, Doctors without Borders), Khayelitsha, Cape Town, South Africa; 2 Department of Medical Microbiology, University of Cape Town, Cape Town, South Africa; 3 City of Cape Town Department of Health, Cape Town, South Africa; 4 Centre for Infectious Disease Epidemiology and Research, University of Cape Town, Cape Town, South Africa; 5 Human Sciences Research Council, HIV/AIDS, STIs and TB programme, Cape Town, South Africa; National Institute of Allergy and Infectious Disease, UNITED STATES

## Abstract

**Background:**

A community based drug resistant tuberculosis (DR-TB) program has been incrementally implemented in Khayelitsha, a high HIV and TB burden community in South Africa. We investigated loss from treatment (LFT), and post treatment outcomes of DR-TB patients in this setting.

**Methodology:**

LFT, defined as interruption of treatment for ≥2 consecutive months was assessed among patients initiating DR-TB treatment for the first time between January 2009 and July 2011. Patients were traced through routine data sources to identify those who subsequently restarted treatment and those who died. Additional information on patient status and survival after LTF was obtained from community DR-TB counselors and from the national death registry. Post treatment outcomes were observed until July 2013.

**Results:**

Among 452 patients initiating treatment for the first time within the given period, 30% (136) were LFT, with 67% retention at 18 months. Treatment was restarted in 27 (20%) patients, with additional resistance recorded in 2/25 (8%), excluding two with presumed DR-TB. Overall, 34 (25%) patients died, including 11 who restarted treatment. Males and those in the age category 15-25 years had a greater hazard of LFT; HR 1.93 (95% CI 1.35-2.75), and 2.43 (95% CI 1.52-3.88) respectively. Older age (>35 years) was associated with a greater hazard of death; HR 3.74 (1.13- 12.37) post treatment. Overall two-year survival was 62%. It was lower (45%) in older patients, and was 92% among those who received >12 months treatment.

**Conclusion:**

LFT was high, occurred throughout the treatment period and was particularly high among males and those aged 15-25 years. Overall long term survival was poor. High rates of LFT should however not preclude scale up of community based care given its impact in increasing access to treatment. Further research is needed to support retention of DR-TB patients on treatment, even within community based treatment programs.

## Introduction

Multidrug resistant tuberculosis [(MDR-TB), defined as *Mycobacterium tuberculosis (M*.*tb)* isolates that are resistant to at least both isoniazid and rifampicin] [1], continues to increase in many TB endemic settings [2–5]. Treatment success rates (cure or treatment completion) remain poor with 48% treatment success globally and 28% of patients reported as lost to follow up or having no outcome, in 2012 and 2013 [2, 3]. Access to treatment for MDR-TB also remains low with an estimated 28% case detection rate globally, and less than 20% of all estimated cases reported to be on treatment in 2012 [3]. A viable model for increasing MDR-TB case detection and scaling up access to care is community-based MDR-TB care which has also been successfully demonstrated in high HIV settings [3, 6–10]. Reported patient outcomes from community based programs, although limited, have to date been similar to those from hospital based programs [11].

In Khayelitsha, a high TB and HIV burden townshipin South Africa, a community-based drug resistant TB [(DR-TB), defined as any rifampicin resistance] program, implemented from 2007 has substantially increased case detection, treatment initiation rates, and reduced the time to treatment initiation [8]. We report on patients who were lost from treatment (LFT), defined in our study as those who interrupted treatment for two or more consecutive months. Our objectives were to investigate LFT, and post treatment outcomes (return to treatment and survival) among DR-TB patients in this setting.

## Methods

### Setting

The patients analyzed were treated in the Khayelitsha decentralized community-based DR-TB program, which is a partnership between the City of Cape Town, the Western Cape Provincial government and Médecins sans Frontières (MSF) [12]. Khayelitsha sub-district is situated 40 km outside Cape Town, in South Africa. The population is estimated at 400 000, 55% of whom live in informal dwellings [13]. 62% of residents 15–64 years were employed in 2011 [13]. The sub-district is served by 10 Primary Health Care (PHC) clinics and one district hospital. A 2008 survey estimated the DR-TB notification rate at 51/100,000/year [14]. In 2009, the TB notification rate was estimated at 1,500 per 100,000 people per year, with antenatal HIV prevalence estimated at 30% [12].

The Khayelitsha DR-TB program implemented from late 2007 has previously been described in detail [8, 12]. In summary an existing TB program at primary care level was enhanced to support care for DR-TB patients by adding the following elements; i) training of healthcare workers (HCWs)(nurses, doctors, counselors, social workers) in managing patients with suspected and confirmed DR-TB, (MSF has a dedicated Medical officer who assists HCWs in managing patients with DR-TB, ii) individualized DR-TB counseling for all newly diagnosed patients, iii) assisting patients to access social assistance iv) enhanced programme supervision and evaluation, v) strengthening and supporting TB infection control mechanisms in health care facilities, vi) establishing a locally based sub-acute in-patient facility for patients to receive support (when needed) while remaining close to their families, vii) specialist DR-TB paediatric outreach services, viii) audiometry screening for early detection of hearing loss among DR-TB patients [12], since aminoglycosides, key drugs in the treatment regimens can cause hearing impairment and loss [15, 16].

### DR-TB diagnosis and treatment

Drug susceptibility testing (DST) which was initially only available for TB cases considered at high risk of DR-TB, (defined as patients previously treated for TB, those not responding to first-line TB treatment, close contacts of patients with DR-TB, healthcare workers, mine workers and those with a prison history), was expanded to all individuals with suspected TB with the introduction of Xpert MTB/Rif [16, 17] from late 2011. Before the introduction of Xpert MTB/Rif, DST for rifampicin and isoniazid was undertaken using a Line-Probe Assay (LPA, Hain Lifesciences, Nehren, Germany) conducted on positive *M*.*tb* positive cultures [18]. Second line (resistance to a flouroquinolone and/or a second line injectable agent) DST was conducted on solid media. Since late 2011, all Xpert MTB/Rif results are confirmed by LPA, with second line DST conducted on solid media.

Treatment regimens used in the programme are generally in line with the World Health Organization (WHO), South African DR-TB treatment guidelines [15, 16], and are tailored according to second-line DST results, with further adjustment when treatment appears to be failing. The standard treatment regime includes kanamycin, ethambutol, ethionamide, pyrazinamide, terizidone, moxifloxacin [8, 12]. Additional drugs are capreomycin, para-aminosalicylic acid (PAS), high-dose isoniazid (INH), clofazimine and linezolid [8, 12]. Treatment for MDR-TB consists of a 6 month intensive phase with 5 drugs that include an injectable agent given for minimum of 6 months, followed by an 18 month continuation phase on oral agents [16]. Culture conversion is defined as two consecutive negative cultures, taken at least 30 days apart [16]. Treatment is given for at least 18 months after culture conversion [16]. Treatment outcome definitions follow WHO and the South African DR-TB treatment guidelines [15, 16].

### Population and data collection

This was a retrospective analysis of routinely collected DR-TB program data. Patients normally resident in Khayelitsha who were newly diagnosed and treated for DR-TB between January 2009 and July 2011 were eligible for inclusion. Patients who were transferred out during treatment were excluded. Survival after LFT was determined passively by surveillance of DR-TB clinic registers to identify patients who restarted treatment, and by conducting regular cross linkage of civil registration numbers with the national death registry to determine deaths and dates of death where registration numbers were available. For those without civil registration numbers, information was obtained from DR-TB counselors who are highly active in local clinics and in the community, and become aware of mortality among DR-TB patients, patients who stop taking DR-TB treatment but continue attending local clinics for other conditions, and those who move out of the area.

Ethics approval for evaluation of the Khayelitsha DR-TB program was obtained from the University of Cape Town Research Ethics Committee (Ref 540/2010).

### Definition of lost from treatment (LFT), and lost to follow up (LTFU)

In the 2013 WHO revised TB definitions and reporting framework [1], patients who do not start treatment or whose treatment is interrupted for two or more consecutive months (previously classified as defaulters) are classified as lost to follow up. For this analysis, we defined patients who interrupted treatment for two or more consecutive months *as lost from treatment* (LFT), and those for whom we had no further information regarding treatment and/or mortality beyond the last known date of treatment as *lost to follow up* (LTFU). This is based on the reality that many patients stop DR-TB treatment but may remain in care for other conditions.

### Data analysis

The data were analysed using Stata version 12.0 (Stata Corp, College Station, TX, USA). Medians were used to summarize non-normally distributed continuous variables, and these were compared using the Mann–Whitney test. The χ^2^ test was used to compare proportions. Patient status (return to treatment and survival) post LFT was assessed until July 2013. Univariate and multivariate cox regression was used to investigate factors associated with duration of treatment before LFT (this analysis excluded patients who died without being LFT), and with time to mortality from date of LFT. The factors tested were, age at the time of DR-TB diagnosis, gender, HIV status, resistance pattern, place of treatment initiation (primary care clinic, sub-acute facility, or hospital), completion of all recommended counseling sessions and culture conversion status at four months of treatment, for duration of treatment before LFT. For survival post treatment (calculated from the date of LFT, which was defined as the last treatment date), we assessed the duration of treatment before LFT, age at the time of DR-TB diagnosis, gender, culture conversion status at LFT, and resistance pattern. Factors that were statistically significant (p< 0.05) on univariate analysis were included in multivariate analyses. A sensitivity (multivariate) analysis including selected factors with p< 0.1 in univariate analysis was also conducted. Life Table analyses were used to calculate patient retention on treatment over time and survival post treatment. The log rank test as used to compare survival proportions.

## Results

### Patient demographics

452 patients who started DR-TB treatment for the first time between January 2009 and July 2011 were included. Among these 48% were successfully treated (160 cured and 55 completed treatment), 30% (136) were LFT, 5% (22) were classified as failure of treatment and 17% (79) died during treatment. The median age at diagnosis was 32 years, with similar proportions of males and females initiating treatment (49% and 51% respectively), Table 1. Approximately two thirds of the patients were HIV infected. The majority (69%) of patients initiated treatment at their primary care clinic. Second line DST results were available for 368 patients (81%), and among these 20 (4.4%) had XDR-TB (defined as resistance to a flouroquinolone and at least one second-line injectable agent (kanamycin or capreomycin in our setting), in addition to multidrug resistance [1], and 38 (8.4%) pre- XDR-TB (defined as isolates multidrug resistance and resistance to either a flouroquinolone or an injectable agent, but not both).

**Table 1:** Demographic characteristics of patients who started treatment between January 2009 and July 2011 who had treatment outcomes by July 2013 (N = 452).

Characteristic	n (%)
**Age at diagnosis (years)**	32 (IQR 24.4–38.4)
**Gender**: Female	230 (51)
**HIV status**	
Negative	135 (30)
Positive	307 (68)
Unknown	10 (2)
**Treatment initiation site**	
PHC Clinic	314 (69)
Sub-acute facility	58 (13)
Hospital	73 (16)
Other	7 (2)
**Counselling sessions**	
Completed	287 (63)
Not completed	165
**>40 DR-TB patients at the clinic (number of clinics)**	
Yes	4 (40)
No	6
**Resistance pattern**	
Rifampicin mono resistance	90 (20)
Presumed DR-TB	32 (7)
MDR no 2ndline resistance	238 (53)
MDR plus 2ndline resistance	58 (13)
MDR 2ndline resistance unknown	34 (8)

PHC-Primary Health Care

MDR- multidrug resistance

### LFT over time and factors associated with LFT

**Fig 1:**
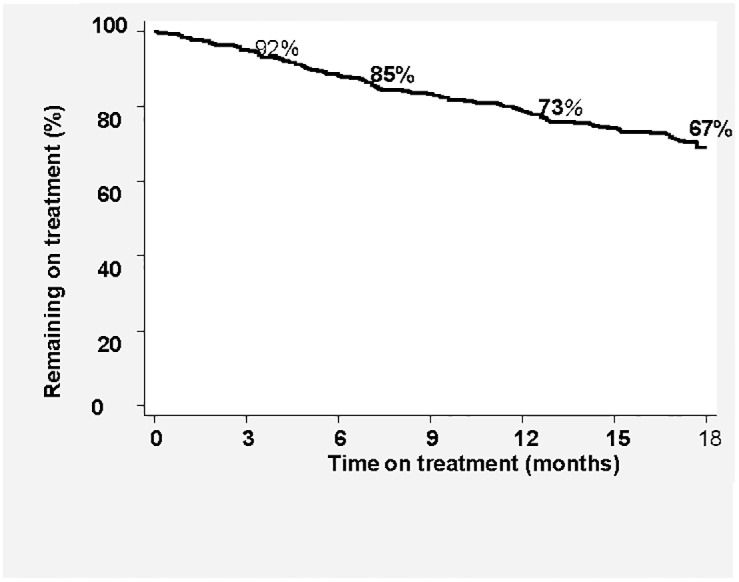
Retention on treatment: Patients remaining on treatment over the course of treatment (n = 393).

Seventy-nine patients died during treatment (without being LFT), and were excluded from this analysis. Median treatment duration before LFT was 7.1 months (IQR 3.6–12.3). Eight percent of patients (37/452) were LFT in the first 3 months of treatment, with 73% and 67% retention at 12 and 18 months of treatment respectively, Fig. 1. In univariate analysis, patients aged 15–25 years, and males were more likely to be LFT, Table 2. These factors remained significant in multivariate analysis, with those aged 25–35 years also having a significantly increased hazard of LFT [HR 1.57 (95%CI 1.02–2.42)]. On inclusion of culture conversion at four months in multivariate analysis, the findings remained unchanged with males and those aged 15–25 years significantly more likely to be LFT, Table 2.

**Table 2:** Association of factors with time to loss from DR-TB treatment—2009–2013 (n = 373*).

Variable	Univariate analysis		Multivariate analysis		Multivariate analysis^	
	HR (95% CI)	p value	HR (95% CI)	p value	HR (95% CI)	p value
**Age**						
0–15 years	0.94 (0.45–1.95)	0.12	1.02 (0.49–2.11)	0.97	1.07 (0.25–4.57)	0.93
>15–25 years	2.20 (1.38–3.50) ^#^	0.001^#^	2.43 (1.52–3.88) ^#^	0.001^#^	2.48 (1.37–4.47)	0.003^#^
>25–35 years	1.40 (0.92–2.14)	0.87	1.57 (1.02–2.42) ^#^	0.04^#^	1.66 (0.97–2.86	0.07
>35 years	Ref					
**Gender:** Male	1.79 (1.26–2.53) ^#^	0.001^#^	1.93 (1.35–2.75) ^#^	<0.001^#^	2.08 (1.31–3.28)	0.002^#^
**HIV positive**	0.98 (0.56–1.15)	0.24				
**Resistance pattern**						
Rifampicin mono resistance	ref					
MDR no 2ndline resistance	0.96 (0.64–1.45	0.85				
MDR plus 2ndline resistance	0.71 (0.34–1.50)	0.37				
MDR 2ndline resistance unknown	0.71 (0.33–1.54)	0.38				
Presumed DR-TB	0.59 (0.25–1.42)	0.24				
**Treatment initiation site**						
PHC Clinic	ref					
Hospital	0.69 (0.39–1.22)	0.21				
Sub-acute facility	0.86 (0.49–1.50)	0.60				
Counselling sessions completed	0.83 (0.55–1.26)	0.94				
Culture conversion at 4 months	2.07 (0.90–4.77)	0.09			2.14 (0.93–4.94)	0.07

* 79 patients who died while on treatment were excluded from this analysis

^#^statistically significant

^ Sensitivity analysis-Multivariate analysis including Culture conversion status at 4 months of treatment

### Return to treatment and survival post LFT

**Fig 2:**
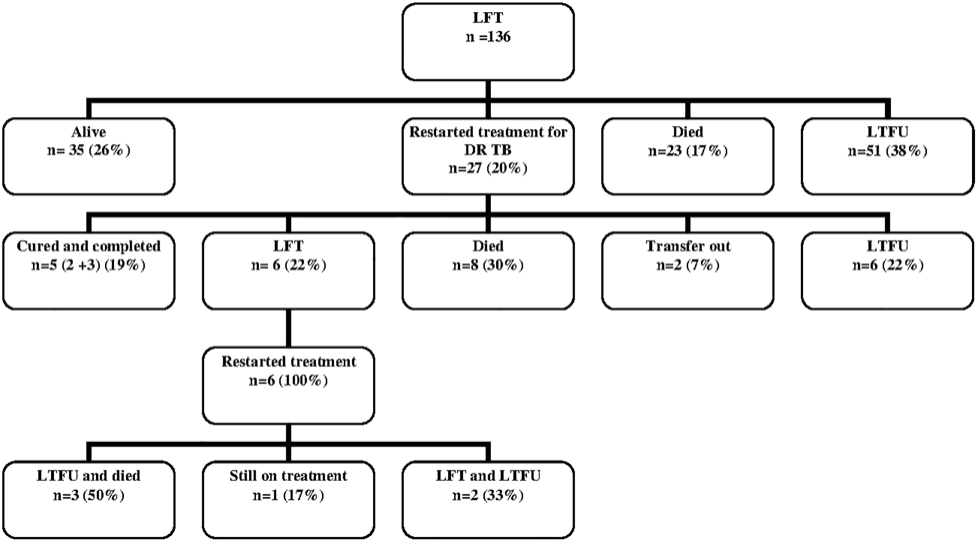
Post treatment outcomes of the patients lost from DR-TB treatment (January 2009-July 2013).

Fig. 2 shows the status of patients who were LFT at the end of our period of observation. No additional information (beyond the last day of treatment), was available for 51 of the 136 patients, after initial recording of LFT. The median period of observation for the remaining 85 patients was 25 months (IQR 10–37 months). Thirty-five patients [35/136 (26%)] were reported to be alive at the end of observation. Twenty-seven (27/136 (20%)), were known to have restarted treatment during this period with the median time to restarting treatment at 6 months (IQR 4.7–13.8). Five (19%) of these 27 patients were then successfully treated, 8 (30%) died while on treatment, 6 (22%) were then LTFU and a further 6 (22%) were LFT again (median duration of treatment 7.3 months (IQR 2.2–13.8)). The median age of the six patients who repeatedly discontinued treatment was 27 years (IQR 22.9–33.7); four were female and three were HIV positive. Four of the total 34 patients (12%) known to have died had second line resistance (1 pre-XDR-TB and 3 XDR-TB). Patients who died without returning to treatment were significantly older than those who returned and those reported to be alive (p = 0.001) at the end of the observation period. Resistance profiles for 25 of the 27 patients who returned to treatment are shown in S1 Table.

### Survival post treatment

**Fig 3:**
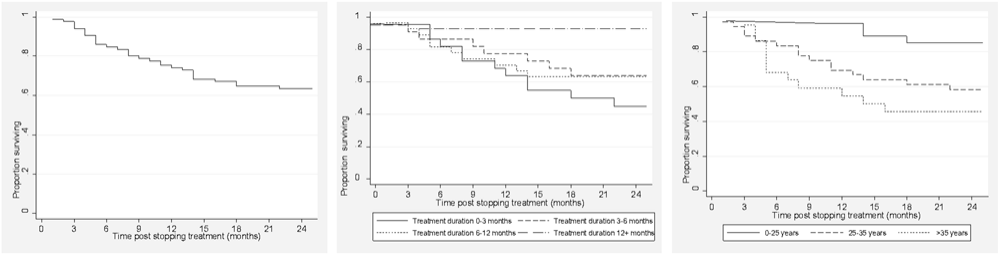
Survival post treatment: Overall, Duration of treatment before LFT, and Age at DR-TB diagnosis.

Survival post was assessed in 85 of the 136 patients (Table 2) with some information after the initial LFT. In univariate analysis the age categories 25–35 years and >35 years were associated with a greater hazard of death, while the hazard was lower in those treated for >12 months before LFT, Table 3. In multivariate analysis the association only remained significant for those aged >35 years, Table 3. Sensitivity analysis including the resistance pattern variable in multivariate analysis showed an increased hazard of death for the age categories 25–35 years and >35 years, with a lower hazard among those with no second line resistance, Table 3. Overall two year survival post treatment was 62%, and was 45% for those >35 years (p = 0.01), Fig. 3. Two-year survival in those with >12 months of treatment was 92%. Two-year survival for patients with less than 6 months of treatment was 52%; significantly lower than for those with >12 months of treatment (p = 0.04).

**Table 3:** Survival post treatment: Factors associated with time to mortality post treatment.

Variable	Univariate analysis		Multivariate analysis n = 66		Multivariate analysis^+^ n = 62	
	(n)					
	HR (95% CI)	p value	HR (95% CI)	p value	HR (95% CI)	p value
**Length of treatment (months)**	(n = 82)					
0–3	ref					
3–6	0.61 (0.25–1.55)	0.30	0.91 (0.32–2.62)	0.87	0.83 (0.23–2.97)	0.78
6–12	0.64 (0.28–1.49)	0.30	0.97 (0.28–3.32)	0.95	0.81 (0.18–3.68)	0.79
12+	0.11 (0.10–0.77)	0.03^#^	0.21 (0.02–1.97)	0.17	0.16 (0.01–1.78)	0.14
**Age (years(**	(n = 82)					
0–25	ref					
>25–35	3.27 (1.07–9.93) ^#^	0.04^#^	3.09 (0.96–9.90)	0.06	3.49 (1.06–11.47) ^#^	0.04^#^
>35	5.08 (1.63–15.79) ^#^	0.01^#^	3.74 (1.13–12.37) ^#^	0.03^#^	3.93 (1.04–14.94) ^#^	0.04^#^
**HIV positive**	(n = 79)					
	0.95 (0.45–2.0)	0.89				
**Gender:** Male	(n = 82)					
	1.47 (0.69–3.15)	0.32				
**Converted at LFT** **	(n = 66)					
	0.45 (0.21–1.00)	0.05	0.58 (0.20–1.72)	0.33	0.52 (0.14–1.94)	0.33
**Resistance pattern**	(n = 74)					
Rifampicin mono resistance	ref				Ref	
MDR no 2ndline resistance	0.50 (0.23–1.09)	0.08			0.35 (0.12–0.97) ^#^	0.04^#^
MDR plus 2ndline resistance	1.11 (0.31–3.97)	0.88			1.20 (0.21–6.93)	0.84

^#^statistically significant

^+^Sensitivity analysis-Multivariate analysis including Resistance pattern

**LFT Lost from treatment

## Discussion

A high proportion of patients initiating treatment in the Khayelitsha community based DR-TB program were LFT. LFT occurred early and persisted throughout the treatment period. Being male and of younger age (15–25 years) were significantly associated with LFT. Overall survival among these patients was relatively poor, but still above that reported for untreated DR-TB patients [19]. Survival was lower again among patients older than 35 years, but there was a trend for improved survival among patients with a longer duration of treatment. Survival two years post LFT was 92% among the small group of patients who had receiving at least 12 months of treatment.

The proportion of patients LFT in this setting is higher than pooled estimates reported in published systematic reviews and meta-analyses, including one study that only analysed outcomes from community based DR-TB programs [11, 20, 21]. Our findings are however comparable to those from Mumbai India (26%), but differ from findings in other settings within South Africa, in Europe and parts of Asia [22–28]. Between 2000 and 2004, the default (LFT in our study) rate in HIV infected and uninfected patients in 8 provinces across South Africa was approximately 21%, equivalent to that found in a high HIV setting in KwaZulu Natal between 2000 and 2003 [23, 24]. However in an analysis of treatment outcomes in the West Coast/Winelands district for the period 1992 to 2002, the default proportion peaked at 40% in 1997 [22]. In Uzbekistan, Latvia, and New Delhi reported rates have been below 20% [26–28]. It should be noted that in many of these settings treatment is centralized, or where decentralized, this is limited, thus only a low proportion of diagnosed patients start treatment [3, 4], in contrast to our setting where over the period 2008–2011, 86% of all patients diagnosed with DR-TB started treatment [8]. Thus, it is likely that we had many patients who would not have received treatment in a centralized model of care, who may potentially be more likely to be LFT. Our sample was also larger than reported in the majority of community based programs reporting lower levels of LFT [6]. Nonetheless, we have previously shown that treatment success in our setting is comparable to that in other high HIV settings [8].

Our findings highlight the difficulty of patient retention on long DR-TB treatment regimens even in a relatively well resourced program. A systematic review by Toczek *et al* showed that engagement of community health workers (CHWs) as Directly Observed Therapy (DOT) providers, provision of DOT throughout the treatment period, patient education and smaller cohort sizes (<100 patients), are associated with lower default rates [20]. In the Khayelitsha program patients receive extensive education, long term support and DOT is provided throughout the treatment period at the local clinic by nursing staff:- and yet LFT was high suggesting that other factors could be driving LFT.

Males and young age were associated with a greater hazard of LFT, consistent with published literature. This could be associated with high risk taking behavior in young males [29]. Males also access health services less frequently than females and adolescents included in this age category have shown poor adherence to chronic medication, both possible explanations for our findings [30, 31]. Khayelitsha experiences in-migration from rural areas and internal migration within the township. Males and young people migrate for school and employment opportunities [32] and may out-migrate if these opportunities do not arise, when employment ends, or to receive care from family during illness. Current patient support and counseling approaches may therefore not be effective in this population group. Research into strategies targeting this population group is therefore needed. Khayelitsha is also a poor township with high rates of unemployment and high alcohol and substance abuse [13, 33], which have both been associated with LFT [34], and require support beyond TB programs.

Absence of culture conversion during the intensive phase and severity of resistance patterns (pre-XDR-TB and XDR-TB) which have been associated with a lower risk of LFT [26, 35] in centralized models of care did not show statistically significant associations in our analysis. However early culture conversion (conversion at four months of treatment), showed a high hazard ratio (HR 2.14, p = 0.07), which although not reaching statistical significance, suggests that it could be associated with LFT in our population. Potentially, patients stop treatment when they start to improve clinically, given the poor tolerability of DR-TB treatment [15]. Indeed, drug toxicity and treatment fatigue were probably significant underlying factors for patients stopping treatment despite extensive counseling and support. Current regimens have severe side effects, comprise a large amount of tablets, include a painful injectable agent, and have to be taken for a long period [15]. DR-TB patients in Armenia reported drug side effects and the long duration of treatment as major factors related to LFT [35]. In India, co-infected patients described the DR-TB drug side effects as ‘*worse than the illness itself’* [36]. A fifth of the patients who stopped treatment early subsequently returned to treatment. This may have been prompted by clinical deterioration, or “some recovery” from the drug side effects. However, we had no data to explore this further in the present analysis.

Overall, more than 60% of patients survived for at least two years after stopping treatment. These patients had received treatment for a median period of 7 months and most were HIV infected. Given disease progression among untreated active disease, it is possible that many of those surviving for this duration no longer had active disease and may not have been infectious [19, 37]. High rates of LFT are often considered as an indicator of poor program performance, with suggestions that such programs are worse than none at all [38]. This is based on the assumption that LFT is associated with high levels of resistance amplification. Although our sample was too small for meaningful conclusions in our population in this regard, (See S1 Table), it should however be noted that the contribution to ongoing transmission from patients who do not complete treatment is most likely minimal in comparison to transmission from the large burden of undiagnosed and untreated cases in high burden settings.

Patient LFT is inherent in DR-TB treatment programs given currently recommended lengthy and arduous treatment regimens [15]. In the Khayelitsha program innovative interventions to reduce patient LFT now include i)piloting patient-managed treatment in the continuation phase with support from CHWs, and ii) early identification and support for patients who interrupt treatment and may therefore be at risk of LFT later on. Cash incentives which have improved adherence in patients with drug sensitive TB [39, 40], may also be effective in DR-TB as suggested by findings from a national cash incentive program for DR-TB patients in Ecuador, where introduction of a monthly cash incentive resulted in a 2.8 fold decrease in the one year default rate [41]. In Khayelitsha, patients are supported in accessing state provided social grants that patients diagnosed with TB, who are unable to continue working or are unemployed are eligible to receive. The high LFT in our supportive setting further highlights the need for less toxic and shorter drug regimens as key to improving patient retention on treatment and achieving better outcomes [42].

Our analysis had several limitations. Firstly we could only assess risk factors based on routinely collected data; hence our assessment of risk factors for LFT is limited since we could not include socio-behavioural and clinical characteristics in our analysis. LFT is likely determined by a complex interplay between socio-demographic, behavioural, economic, clinical, bacteriological and programmatic factors some of which cannot be easily assessed with routine data. Post treatment outcomes were determined passively with no active follow up of patients, therefore patient status could have been misclassified, particularly among patients not reported to have died, who were then regarded as LTFU at the end of observation; therefore our observed 62% two-year survival could be falsely inflated.

LFT in this community based DR-TB program while high is comparable to that in a similar setting in India, and to that in some centralized programs in high HIV and TB burden settings. Overall two-year survival post LFT was relatively poor. High rates of LFT should not preclude treatment scale up, since community based treatment is an effective strategy for increasing access to treatment. Further research into interventions for supporting patients undergoing DR-TB treatment even in community based programs, and improved shorter treatment regimens are urgently needed.

## Supporting Information

S1 Table *Mycobacterium tuberculosis* resistance profiles* of patients who returned to treatment (n = 25). (DOCX)

## References

[1] World Health Organization. Definitions and reporting framework for tuberculosis—2013 revision. 2013. Available: http://apps.who.int/iris/bitstream/10665/79199/1/9789241505345_eng.pdf

[2] World Health Organization. Global tuberculosis report 2012. 2012. Available: http://apps.who.int/iris/bitstream/10665/75938/1/9789241564502_eng.pdf.

[3] World Health Organization. Global tuberculosis report 2013. 2013. Available: http://apps.who.int/iris/bitstream/10665/91355/1/9789241564656_eng.pdf.

[4] World Health Organization. Countdown to 2015 Global Tuberculosis Report 2013 Supplement. 2013 Available: http://apps.who.int/iris/bitstream/10665/91542/1/WHO_HTM_TB_2013.13_eng.pdf

[5] World Health Organization. Multi drug resistant tuberculosis 2013 update. 2013. Available: www.who.int/tb/challenges/mdr/MDR_TB_FactSheet.pdf.

[6] Heller T, Lessells RJ, Wallrauch CG, Bärnighausen T, Cooke GS, Mhlongo L, et al Community-based treatment of drug-resistant tuberculosis in rural KwaZulu-Natal, South Africa. *Int J Tuberc Lung Di*s. 2010; 4: 420–426. 20202299

[7] Brust JC, Shah NS, Scott M, Chaiyachati K, Lygizos M, van der Merwe L, et al Integrated, home-based treatment for MDR-TB and HIV in rural South Africa: an alternate model of care. *Int J Tuberc Lung Dis*. 2012; 8:998–1004. 10.5588/ijtld.11.0713 22668560 PMC3390442

[8] Cox HS, Hughes J, Daniels J, Azevedo V, McDermid C, Poolman M, et al Community-based treatment of drug-resistant tuberculosis in Khayelitsha, South Africa. *Int J Tuberc Lung Dis*. 2014; 4: 441–8. 10.5588/ijtld.13.0742 24670700

[9] Bassilli A, Fitzpatrick C, Qadeer, Fatima R, Floyd K, Jaramillo E. A systematic review of the effectiveness of hospital and ambulatory- based management of multidrug resistant tuberculosis. *Am J Trop Med Hyg*. 2013; 89: 271–280. 10.4269/ajtmh.13-0004 23926140 PMC3741248

[10] Fitzpatrick C, Floyd K. A systematic review of the cost and cost effectiveness of treatment for multidrug resistant tuberculosis. *Pharmacoeconomics* 2012; 30:63–80. 10.2165/11595340-000000000-00000 22070215

[11] Weiss P, Chen W, Cook VJ, Johnston JC. Treatment outcomes from community-based drug resistant tuberculosis treatment programs: a systematic review and meta-analysis. *BMC Infect Dis*. 2014; 14: 333 10.1186/1471-2334-14-333 24938738 PMC4071022

[12] Medecins sans Frontieres. Scaling up diagnosis and treatment of drug-resistant tuberculosis in Khayelitsha, South Africa. 2011. Available: http://www.msfaccess.org/sites/default/files/MSF_assets/TB/Docs/TB_report_ScalingUpDxTxKhaye_ENG_2011.pdf.

[13] City of Cape Town. City of Cape Town–2011 Census Suburb Khayelitsha. 2013. Available: http://www.capetown.gov.za/en/stats/2011CensusSuburbs/2011_Census_CT_Suburb_Khayelitsha_Profile.pdf

[14] Cox HS, McDermid C, Azevedo V, Muller O, Coetzee D, Simpson J, et al Epidemic Levels of Drug Resistant Tuberculosis (MDR and XDR-TB) in a High HIV Prevalence Setting in Khayelitsha, South Africa. PLoS ONE 2010; 5(11): e13901 10.1371/journal.pone.0013901 21085569 PMC2981525

[15] World Health Organization Guidelines for the programmatic management of drug-resistant tuberculosis: Emergency update 2008. Geneva. 2008. Available: http://whqlibdoc.who.int/publications/2008/9789241547581_eng.pdf 23844450

[16] National Department of Health, South Africa, Pretoria. Management of Drug resistant Tuberculosis. Policy guidelines, 2011. Available: http://www.doh.gov.za/docs/policy/2011/policy_TB.pdf.

[17] World Health Organization. Stop TB Department. Update: Implementation and roll-out of Xpert MTB/RIF. 2012. Available: http://www.stoptb.org/wg/gli/assets/documents/Xpert%20MTB-RIF%20UPDATE%20February%202012.pdf.

[18] Barnard M, Albert H, Coetzee G, O’Brien R, Bosman M E. Rapid molecular screening for multidrug-resistant tuberculosis in a high-volume public health laboratory in South Africa. *Am J Respir Crit Care Med*. 2008; 177: 787–792. 10.1164/rccm.200709-1436OC 18202343

[19] Gandhi NR, Shah NS, Andrews JR, Vella V, Moll AP, Scott M, et al HIV co-infection in multidrug- and extensively drug-resistant tuberculosis results in high early mortality. *Am J Respir Crit Care Med*. 2010; 1: 80–6. 10.1164/rccm.200907-0989OC 19833824

[20] Toczek A, Cox H, Cros P, Cooke G, Ford N. Strategies for reducing treatment default in drug resistant tuberculosis: systematic review and meta-analysis. *Int J Tuberc Disease* 2013; 7: 299–307. 10.5588/ijtld.12.0537 23211716

[21] Johnston JC, Shahidi NC, Sadatsafavi M, Fitzgeral JM. Treatment outcomes of multidrug resistant tuberculosis: A systematic review and meta-analysis. *PLoS ONE*. 2009; 4 (9): e6914 10.1371/journal.pone.0006914 19742330 PMC2735675

[22] Shean KP, Wilcox PA, Siwendu SN, Laserson KF, Gross L, Kammerer S, et al Treatment outcome and follow up of multidrug resistant tuberculosis patients, West Coast/Winelands South Africa 1992–2002. *Int J Tuberc Lung Dis*. 2008; 12:1182–1189. 18812049

[23] Farley JE, Ram M, William P, Waldman S, Cassell GH, Chaisson RE, et al Outcomes of Drug Resistant Tuberculosis (MDR-TB), among a cohort of South African Patients with high HIV prevalence. *PLoS ONE*. 2011; 6(7): e20436 10.1371/journal.pone.0020436 21799728 PMC3142109

[24] Burst JC, Gandhi NR, Carrara H, Osburn G, Padayatchi N. High treatment failure and default rates for patients with multi drug resistant tuberculosis in KwaZulu Natal, South Africa, 2000–2003. *Int J Tuberc Lung Dis*. 2010; 14: 413–419. 20202298 PMC3005763

[25] Isaakidis P, Cox HS, Varghese B, Montaldo C, Da Silva E, Mansoor H et al Ambulatory multidrug resistant tuberculosis treatment in a cohort of HIV infected patients in a slum setting in Mumbai, India. *PLoS ONE*. 2011; 9(12): e28066. 10.1371/journal.pone.0028066 PMC3228724 22145022

[26] Lalor MK, Greig J, Allamuratova S, Althomsons S, Tigay Z, Khaemraev A, et al Risk factors associated with default from multi and extensively resistant tuberculosis treatment, Uzbekistan: A retrospective cohort analysis. *PLoS One*. 2013; 8(11): e78364 10.1371/journal.pone.0078364 24223148 PMC3819387

[27] Liemane V, Riekstina V, Holtz TH, Zaroviska E, Skripconoka V, Thorpe LE, et al Clinical outcomes of individualized treatment of multidrug resistant tuberculosis in Latvia: a retrospective cohort study. *Lancet*. 2005; 365: 318–326. 15664227 10.1016/S0140-6736(05)17786-1

[28] Liu CH, Li L, Chen Z, Wang Q, Hu YL, Zhu B, et al Characteristics and treatment of patients with MDR and XDR tuberculosis in a TB referral hospital Beijing: a 13 year experience. *PLoS ONE*. 2011 6(4): e19399 10.1371/journal.pone.0019399 21559362 PMC3084844

[29] Geldenhuys H, Sorsdahl K, Kafaar F, Hatherill M, Hanekom WA, Stein DJ, et al Risky behaviour and psychosocial correlates in adolescents—is there a link with tuberculosis? *African Journal of Psychiatry*. 2011; 14: 383–387. 10.4314/ajpsy.v14i5.6 22183469

[30] Galdas PM, Cheater F, Marshall P. Men and health help-seeking behaviour: literature review. *J Adv Nurs*. 2005; 49: 616–62. 15737222 10.1111/j.1365-2648.2004.03331.x

[31] Letsela L, Ratele K. I am totsi from Sophiatown, you must cure yourself”: Masculinity and health seeking behaviour in South Africa. 2009. Available: http://www.mrc.ac.za/crime/maschealthseek.pdf.

[32] De Azevedo V. (Intersectoral action for health and well-being in Khayelitsha: A health sector perspective. 2013. Available: http://www.hivaids-uwc.org.za/docs/symposia2013/Intersectoral_action_for_health_and_well-being_in_Khayelitsha_A_health_sector_perspective.pdf.

[33] City of Cape Town. Socioeconomic profiling of urban renewal nodes. Khayelitsha and Mitchell’s plain. 2006. Available: http://www.capetown.gov.za/en/stats/CityReports/Documents/Urban%20Renewal%20Programme/URP_Socio-Eco_Report_228200612953_359.pdf.

[34] Holtz TH, Lancaster J, Laserson KF, Wells CD, Thorpe L, Weyer K. Risk factors associated with default from multidrug-resistant tuberculosis treatment, South Africa, 1999–2001. *Int J Tuberc Lung Dis*. 2006; 10:649–55. 16776452

[35] Sanchez- Padilla E, Marquer C, Kalon S, Qayyum S, Hayrapetyan A, Varaine F, et al. Reasons for default from drug resistant tuberculosis treatment in Armenia: a quantitative and qualitative study. *Int J Tuberc Lung Dis*. 2014; 2: 160–167. 10.5588/ijtld.13.0369 24429307

[36] Petros I, Rangan S, Pradhan A, Ladomirska J, Reid T, Kielmann K. (‘I cry every day’: experiences of patients co-infected with HIV and multidrug-resistant tuberculosis. *Trop Med Int Health*. 2013; 9: 1128–33. 10.1111/tmi.12146 23837468

[37] American Thoracic Society/Centers for Disease Control and Prevention/Infectious Diseases Society of America. Controlling tuberculosis in the United States. *Am J Respir Crit Care Med*. 2005; 172: 1169–1227. 16249321 10.1164/rccm.2508001

[38] Chiang CY, Van Deun A, Enarson DA. A poor drug-resistant tuberculosis programme is worse than no programme: time for a change. *Int J Tuberc Lung Dis*. 2003; 6: 714–8. 10.5588/ijtld.12.0989 23575274

[39] Davidson H, Schluger NW, Feldman PH, Valentine DP, Telzak EE, Laufer FN. The effects of increasing incentives on adherence to tuberculosis directly observed therapy. *Int J Tuberc Lung Dis*. 2000; 4: 860–865. 10985655

[40] Lutge E, Lewin S, Volmink J, Friedman I, Lombard C. Economic support to improve tuberculosis treatment outcomes in South Africa: a pragmatic cluster-randomized controlled trial. *Trials*. 2013; 14:154 10.1186/1745-6215-14-154 23714270 PMC3680200

[41] Sripad A, Castedo J, Danford N. Zaha R, Freile C. Effects of Ecuador’s national monetary incentive program on adherence to treatment for drug-resistant tuberculosis. *Int J Tuberc Lung Dis*. 2013; 1: 44–48. 10.5588/ijtld.13.0253 24365551

[42] Médecins sans Frontières. DR-TB drugs under the microscope Sources and prices for drug resistant tuberculosis medicines 3rd edition 2013 Available: http://www.artsenzondergrenzen.nl/pdf/MSF_TB_Report_Drugs_under_the_microscope_2013.pdf.

