# Supplementary material for: Loss from Treatment for Drug Resistant Tuberculosis: Risk Factors and Patient Outcomes in a Community-Based Program in Khayelitsha, South Africa
Source: PLoS One. 2015 Mar 18;10(3):e0118919. doi: 10.1371/journal.pone.0118919 (PMC4364980; doi:10.1371/journal.pone.0118919)
Supplement: S1 Table — (DOCX) [file pone.0118919.s001.docx]

***Resistance profiles on return to treatment***

Resistance profiles for 25 of the 27 patients who returned to treatment are shown in Supplementary in **Table 1**. (Two patients had presumed DR-TB, and were excluded from further analysis of the sub-group). Two (8%) of the 25 remaining patients had additional resistance when they returned to treatment; the first of these patients (patient number 10, **Supplementary File Table 1**) had received 19.1 months of treatment, with one negative culture result, and was then LFT for 2.2 months before restarting treatment. The second patient had received 7.1 months of treatment, with five negative culture results, before LFT (patient number 22), and returned to treatment after 18.6 months. Only one of the 33 patients who culture converted after four months of treatment developed additional resistance. This proportion is probably lower than would be expected since additional drugs beyond those provided under the national guidelines were available for selected patients in Khayelitsha. These findings could suggest that development of additional resistance on returning to treatment is most likely not higher than that which occurs during well supervised DR-TB treatment. However our sample was too small draw meaningful conclusions.

**Supplementary Table 1: *Mycobacterium tuberculosis* resistance profiles* of patients who returned to treatment (n=25)**

| **Patient number** | **Duration of treatment before stopping (months)** | **Resistance profile initiation of treatment** | **Negative sputum sample culture at time of default** | **Resistance profile on return to treatment** | **Time period before returning to treatment (months)** |
| --- | --- | --- | --- | --- | --- |
| 1 | 4.2 | RIF + INH | No | RIF+ INH | 18.4 |
| 2 | 11.8 | RIF + INH | Yes | Culture negative | 1.7 |
| 3 | 4.8 | RIF + INH | Yes | Culture negative | 11.7 |
| 4 | 1.2 | RIF + INH | No | RIF+ INH | 11.2 |
| 5 | 3.1 | RIF | No | RIF | 22.6 |
| 6 | 7.1 | RIF | Yes | Culture negative | 6.4 |
| 7 | 1.9 | RIF | Yes | Culture negative | 8.4 |
| 8 | 2.8 | RIF + INH | No | RIF + INH | 7.5 |
| 9 | 5.9 | RIF + INH | No | RIF + INH | 16.4 |
| *^#^10* | *19.1* | *RIF+ INH* | *Yes* | *RIF+ INH+ Ofx* | *2.2* |
| 11 | 6.7 | RIF+ INH | Yes | Culture negative | 13.6 |
| 12 | 4.4 | RIF + INH | No | RIF + INH | 14 |
| 13 | 0.9 | RIF + INH | No | RIF + INH | 5 |
| 14 | 2.8 | RIF + INH +Ofx | No | RIF + INH | 3.8 |
| 15 | 1.6 | RIF + INH | No | RIF + INH | 5.6 |
| 16 | 8.3 | RIF + INH | Yes | Culture negative | 2.1 |
| 17 | 0.4 | RIF + INH | No | RIF + INH | 6.9 |
| 18 | 0.5 | RIF | No | RIF | 3.6 |
| 19 | 1.2 | RIF + INH | No | RIF + INH | 8.9 |
| 20 | 8.2 | RIF + INH | No | RIF | 4.7 |
| 21 | 12.9 | RIF | No | RIF | 6.9 |
| *^#^22* | *7.1* | *RIF+INH+ Am* | *Yes* | *RIF +INH+ Am+ Ofx* | *18.6* |
| 23 | 4.9 | RIF + INH | No | RIF + INH | 2.7 |
| 24 | 2.8 | RIF + INH | No | RIF + INH | 4.7 |
| 25 | 1.8 | RIF + INH | No | RIF + INH | 3.6 |

*Profiles at initiation of treatment, sputum specimen status and first profiles on return to treatment are shown

**^#^**Cases showing additional resistance on return to treatment)

Culture conversion status defined as two consecutive negative cultures taken at least 30 days apart is not used in this analysis, since some patients in this subgroup were on treatment for less than 60 days. Rif- rifampicin; INH- isoniazid; Am- Amikacin; Ofx- Ofloxacin
